# Freshness-Preserving Non-Interactive Hierarchical Key Agreement Protocol over WHMS

**DOI:** 10.3390/s141223742

**Published:** 2014-12-10

**Authors:** Hyunsung Kim

**Affiliations:** Department of Cyber Security, Kyungil University, Kyungbuk 712-701, Korea; E-Mail: kim@kiu.ac.kr; Tel.: +82-53-600-5621; Fax: +82-53-600-7609

**Keywords:** wireless healthcare, freshness-preserving, non-interactive key agreement, key agreement, bilinear pairing, anonymity, body area network, security

## Abstract

The digitization of patient health information (PHI) for wireless health monitoring systems (WHMSs) has brought many benefits and challenges for both patients and physicians. However, security, privacy and robustness have remained important challenges for WHMSs. Since the patient's PHI is sensitive and the communication channel, *i.e.*, the Internet, is insecure, it is important to protect them against unauthorized entities, *i.e.*, attackers. Otherwise, failure to do so will not only lead to the compromise of a patient's privacy, but will also put his/her life at risk. This paper proposes a freshness-preserving non-interactive hierarchical key agreement protocol (FNKAP) for WHMSs. The FNKAP is based on the concept of the non-interactive identity-based key agreement for communication efficiency. It achieves patient anonymity between a patient and physician, session key secrecy and resistance against various security attacks, especially including replay attacks.

## 1. Introduction

With an aging society, people are interested in health and desire to manage their healthy life by themselves. With the evolution of medical technology and information technology (IT) convergence, it is now possible for people to gather information on their health status anytime and anywhere easily using biometric information acquisition devices over wireless health monitoring systems (WHMSs) [1,2]. Especially, the recent technological advances in sensors, low-power integrated circuits and wireless communications have enabled the design of low-cost, miniature, lightweight and intelligent physiological sensor nodes. These sensors, capable of sensing, processing and communicating one or more vital signs, can be seamlessly integrated into wireless personal area networks (WPANs) or wireless body area networks (WBANs) for WHMSs [3]. A WBAN contains a number of portable, miniaturized and autonomous sensor nodes (in-body or/and on-body nodes) that monitors patients under natural physiological status without constraining their normal activities. The gateway (e.g., personal computer or mobile phone) of the WBAN is responsible for data collection, processing and overall WBAN management. These networks promise to revolutionize healthcare by allowing inexpensive and non-invasive continuous health monitoring with almost real-time updates of medical records via the Internet. On the other hand, the different usage scenarios of WHMSs, which are composed of various ranges, including pre-hospital, in-hospital, ambulatory and in-home monitoring, have resulted in diverse security and privacy concerns [4–8]. Furthermore, due to the sensitive nature of some of the remotely electronically collected patient health information (PHI), combined with the insecure nature of the communication channels, *i.e.*, the Internet, there is a need to prevent unauthorized access to and use of the PHI by both active and passive attackers. Otherwise, failure to do so will not only put a patient's privacy in jeopardy, but also his/her life will be at risk.

The transmitted information must be secured to protect patient privacy. Furthermore, the physician (doctor/nurse), the ubiquitous health (u-Health) server or the electronic health record (EHR) database that collects and treats the data must be confident that the data are unaltered and indeed originated from the specified patient [9]. The major challenges in WHMSs are security, privacy and robustness. Many security protocols to enhance security, privacy and robustness in WHMSs have been put forward by researchers [10–16]. Huang *et al.*, proposed an identity-based authentication and context privacy preservation scheme in WHMS [10]. They adopted identity-based encryption to protect the confidentiality of PHI. However, Huang *et al.*'s scheme does not provide patient identity privacy and is also weak against password guessing attacks on the physician's side [11,12]. Layouni *et al.*, proposed a privacy protection protocol for remote monitoring of medical care [13]. Their protocol is based on the symmetric encryption and Rivest-Shamir-Adleman (RSA) algorithm to complete the encryption and authentication for the PHI. Various security schemes are proposed separately for the different aspect of security, including Hasque *et al.*'s security scheme for u-Healthcare sensor networks based on the public key cryptosystem in [14], Yang *et al.*'s password-based authentication scheme for healthcare delivery systems in [15] and Mtonga's secure authentication scheme for the WHMS using WBAN in [16]. Mtonga's scheme is based on identity-based cryptography (IBC), the bilinear pairing and the non-interactive identity-based key agreement scheme. Even though he argued that his scheme provides a privacy preserving property, it could not provide session freshness, because the scheme uses the same session key in every session. The focus on this paper is to propose a remedy mechanism for Mtonga's scheme [16], which is only focused on the key agreement.

Since Sakai *et al.*, proposed a non-interactive key agreement protocol based on the bilinear pairing, and researchers have focused on devising protocols for various network environments based on it [17–22]. Guo *et al.*, proposed an efficient and non-interactive hierarchical key agreement protocol applicable to mobile *ad hoc* networks [18]. Guo *et al.*'s protocol is based on the bilinear pairing and satisfies the desired properties mentioned in [19] for the authenticated key agreement protocol of the mobile *ad hoc* networks and tactical networks. However, their protocol could not be applied to wireless sensor networks (WSNs), due to the WSNs' uniqueness. Thereby, there are some research efforts on proposing hierarchical key agreement protocols in WSNs, including Lee *et al.*'s effort, which is a revised version of Guo *et al.*'s protocol for WSNs [20–22]. Lee *et al.*'s protocol is secure against the corruption of any number of nodes at any level in the hierarchy. However, their protocol could not be applied to the WHMS environment due to the WHMSs' unique properties and the different system requirements.

This paper proposes a secure and freshness-preserving non-interactive hierarchical key agreement protocol (FNKAP) for WHMS. The FNKAP is based on the IBC and the non-interactive identity-based key agreement for communication efficiency between any two entities in a WHMS. The FNKAP only requires one round of communication to establish a secure communication channel between any two entities in a WHMS. The FNKAP over a WHMS consists of three parties, including the patient with the WBAN, the u-Health server with the EHR database and the physician. It is based on the IBC to ensure the secure transmission, receiving, storing and access of the PHI. This could ensure the confidentiality of the PHI, which, in turn, is crucial for accurate diagnoses of a patient by his/her respective physician. The FNKAP inherits the advantages of the previous non-interactive key agreement protocols and removes the problems with them, which achieves patient anonymity between a patient and physician, session key secrecy and resistance against replay attacks.

This paper is organized as follows. Section 2 reviews a WHMS configuration and basic security mechanisms. A new FNKAP is proposed over a WHMS in Section 3. Analyses, including correctness, security analysis, functionality and performance analyses, are provided in Section 4. Finally, Section 5 gives a brief conclusion.

## 2. Preliminaries

This section details the system architecture with the threat model in a WHMS and the mathematical background, which can be used as the basic difficulties of the FNKAP [18,22–24].

### 2.1. WHMS Configuration and Threat Model

**Figure 1.**
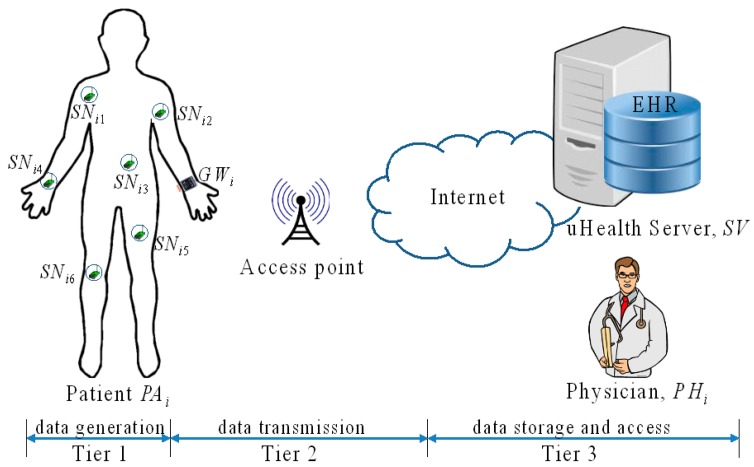
Wireless health monitoring system (WHMS) configuration.

We consider a basic architecture, depicted in Figure 1, consisting of disparate devices and multiple access points. The main parties in our system are sensor nodes *SN_i_*, a gateway *GW_i_* and a back-end server, which are composed of a u-Health server *SV* with the EHR database and attending physicians *PH_i_*.

An at-home patient wears some wireless sensor devices *SN_i_* to read his/her blood pressure, electrocardiogram (ECG), *etc.*, once per second. These readings are periodically communicated to a gateway, *i.e.*, a smartphone or a smart watch, which may be incorporated with a utility to view the physiological readings, and the data stream is uploaded over the Internet to a centralized u-Health server *SV*. Authorized personnel *PH_i_*, such as doctors and nurses, accesses the database directly to diagnose and monitor patients. The data are also of interest to litigators for forensics, to investigate malpractice and assign liabilities. Our aim is to establish a secure channel by setting up a secure session key to cope with tampering throughout the WHMS network, while allowing it to be easily viewed by the legal entities. The WHMS usually consists of three tiers, as follows:

- Tier 1 [*SN_i_*]: This is for sensor nodes, which are wearable or implantable devices placed on the patient's body. This is responsible for the sensing and transmitting of the PHI to the back-end u-Health server.
- Tier 2 [*GW_i_*]: This is the gateway, which is the personal server, and this computer software that could be installed on a personal smart phone or smart watch. It is responsible for the collection of the PHI, as well as pretreatment and communication with the u-Health server *SV* or the attending physicians *PH_i_*.
- Tier 3 [*SV, PH_i_*]: This is the back-end server, *i.e.*, u-Health server, which has the role of private key generator (PKG) and has the EHR database located in the medical institution. It is mainly responsible for key setup, key management, data analysis, data management and data processing. It also includes attending physicians *PH_i_*, because *PH_i_* has a critical role for the patients in the WHMS via *SV* or EHR. The EHR is a database of medical data objects and health-related data managed by health professionals. The EHR is a subset of the electronic medical record maintained by each WHMS and is created and owned by the patient *PA_i_*.

There are many threats to the WHMS. In the FNKAP, we assume that the u-Health server at Tier 3 is trustworthy. However, two communications are unsecure between *SN_i_* on Tier 1 and *GW_i_* on Tier 2 and between *GW_i_* and *SV* on Tier 3. The reasons are that *SN_i_* and *GW_i_* interact over wireless communication and *GW_i_* and *SV* transfer data over the public Internet. The FNKAP considers both active and passive threats for the WHMS. A passive threat involves an attack that attempts to gain access to information without affecting the communication; whereas an active threat attempts to change the communication that it is attacking. Some examples of each type of threat are the replay attack and modification attack. A replay attack captures information sent by an entity and later attempts to reuse (replay) that information in order to gain access to protected data. A modification attack changes the information included in messages being processed between two or more entities.

Furthermore, we consider privacy threats focused on anonymity and PHI data privacy against the insiders of the EHR [23]. Privacy is considered as information relating to an identified or identifiable individual. Privacy threats come in a number of forms, including threats to financial standing, reputation, solitude, autonomy and safety. Intrusion or interruption of an individual's life or activities can threaten the individual's ability to be left alone. Communications may be directed between the initiator and the recipient or additional entities may be involved in packet forwarding, which may interfere with privacy protection goals, as well. Although the additional entities may not generally be considered as attackers, they may all pose privacy threats, because they are able to observe and collect privacy-relevant data. From a privacy perspective, one important type of attacker is a passive attacker, who is an entity that passively observes the entity's communications without the entity's knowledge or authorization. Different kinds of attacks may be feasible at different points in the communications path. A passive attacker could mount surveillance or identification attacks between two communication participants.

### 2.2. Security Mechanisms

For the security mechanisms, including the bilinear map, Diffie–Hellman (DH) problem and non-interactive identity-based key agreement, we need to have some common assumptions as follows. Let *G*_1_ be an additive group of prime order *q* and *G*_2_ be a multiplicative cyclic group of the same order. In reality, *G*_1_ is a subgroup of points on an elliptic curve over Z_q_*, and *G*_2_ is a subgroup of the multiplicative group of a finite field $Z_{q}^{*}k$ for some *k* ∊ Z_q_*. Let *P* denote a generator of *G*_1_.

#### 2.2.1. Bilinear Map

There exists an efficient computable bilinear map *ê*: *G*_1_ × *G*_1_ → *G*_2_, which has the following properties [24]:

- Bilinearity: Given *P* and *Q* in *G*_1_ and *a, b* ∊ Z_q_*, we have *ê*(*a*·*P, b*·*Q*) = (*P, Q*)*^a^*^·^*^b^*.
- Non-degeneracy: *ê*(*P, P*) ≠ 1*_G_*_2_.
- Computability: There exists an efficient algorithm to compute *ê*(*P, Q*) for any *P, Q* ∊ *G*_1._

#### 2.2.2. Diffie–Hellman Problem

There are two DH problems, bilinear DH (BDH) and computational DH (CDH). The BDH problem is to compute *ê*(*P, P*)*^a^*^·^*^b^*^·^*^c^* ∊ *G*_2_ given *P* ∊ *G*_1_ and elements *a*·*P, b*·*P, c*·*P* ∊ *G*_1_ for *a, b, c* ∊ Z_q_*. Computing such a problem is assumed to be hard on {*G*_1_, *G*_2_, *ê*}. The CDH problem is given as (*P, a*·*P, b*·*P*), and computing *a*·*b*·*P* is assumed to be hard.

#### 2.2.3. Non-Interactive Identity-Based Key Agreement

For the non-interactive identity-based key agreement protocol, a central authority first generates two cyclic groups *G*_1_ and *G*_2_ and the bilinear map *ê* to setup the parameters for an IBC. The central authority also chooses a cryptographic collision-free hash function *H*(·):{0,1}* → *G*_1_. It then chooses a secret key *s* ∊ Z_q_* and computes a corresponding public key *P_pub_* = *s*·*P*, where *P* is a generator of *G*_1_. Lastly, it publishes public parameters {*G*_1_, *G*_2_, *ê, P, P_pub_, H*(·)}. For the registered party *i*, the central authority computes a private key *D_i_* = *s*·*H*(*ID_i_*) and sends it to him/her via a secure channel [18,22].

For two clients in the same PKG with identities, *ID*_1_ and *ID*_2_, the shared key is given by *SK* = *ê*(*H*(*ID*_1_), *H*(*ID*_2_))*^s^*, which the party *ID*_1_ computes as *SK*_1,2_ = *ê*(*D*_1_, *H*(*ID*_2_)), and *ID*_2_ computes *SK*_2,1_ = *ê*(*D*_2_, *H*(*ID*_1_)). Clearly, *SK*_1,2_ = *SK*_2,1_ = *SK*.

## 3. Freshness-Preserving Non-Interactive Hierarchical Key Agreement Protocol (FNKAP)

This section proposes a freshness-preserving non-interactive hierarchical key agreement protocol (FNKAP) for WHMSs. The FNKAP is based on the IBC to setup a secure channel between two entities, which is used for the secure communications over WHMSs. This ensures the security of the PHI, which, in turn, is crucial for accurate diagnoses of a patient by his/her respective physician. To achieve patient anonymity, we adopt pseudonyms, which are issued by the PKG to the patient upon successful registration via a secure channel. The FNKAP has three parties, namely the patient with WBAN, the u-Health server with the EHR database and the physician. It falls into three phases, a system initialization phase, physician and patient registration phase and non-interactive key agreement and secure communication phase. The first phase is for setting up the system, and the other two are to register participants, to establish a secure channel and to perform secure communications between the patient and the physician or any two parties in the hierarchy of a key tree in WHMSs.

We assume that all communications between the patient and the EHR, the EHR and the physician and the physician and the patient are carried out over an insecure channel, *i.e.*, the Internet. In the FNKAP, the u-Health server plays the role of the registration server, system parameter generator or trusted authority and the authentication server. Furthermore, it is assumed that the network is formed in a hierarchy; one hop is considered between sensor nodes and a gateway node over the WBAN. We begin this section by discussing a permission hierarchy for the WHMS for the key setup and then detail the phases of the FNKAP.

### 3.1. Permission Hierarchy of WHMS

**Figure 2.**
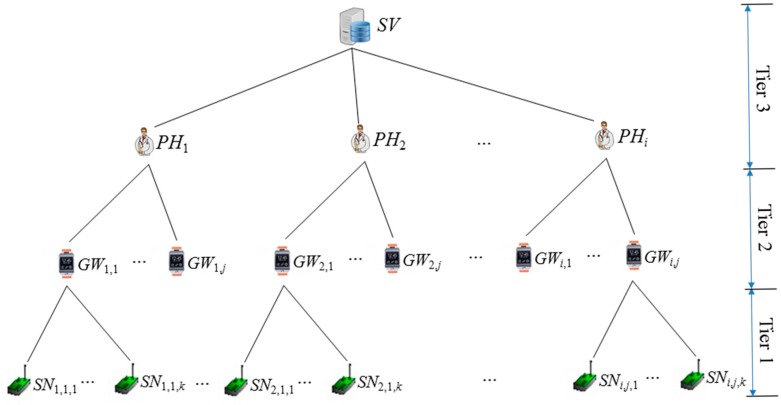
Permission hierarchy of a WHMS.

The FNKAP requires pre-established keys to secure WHMSs. Conceptually, the key setup for the FNKAP is based on a tree permission hierarchy, as shown in Figure 2. The tree is formed by considering permission level of entities of the WHMS, which are *SV, PH_i_, GW_i_*_,_*_j_* and *SN_i_*_,_*_j_*_,_*_d_*. The u-Health server performs the role of the PKG, which puts permission depending on their roles in a hierarchy. The most prominent entity is the u-Health server *SV*, which is classified as Tier 3 in Figure 2, but discrete permissions can be set at a much finer level. Note that the u-Health server could provide different permissions with the EHR depending on the system utilization. It is a natural way to implement a hierarchy, as in Figure 2, so as to reflect the role structure to show the line of authority and responsibility. Conventionally, more privilege is shown toward the top of the tree and less privilege toward the bottom. The privilege of the u-Health server contains the highest permission, which, in turn, contains the role of the others, *PH_i_, GW_i_*_,_*_j_* and *SN_i_*_,_*_j_*_,_*_d_*. Because of the transitive nature of permission hierarchies, *PH_i_* also contains the permission of *GW_i_*_,_*_j_* and *SN_i_*_,_*_j_*_,_*_d_*. Capabilities are granted to entities by their parents. Thus, *SV* grants capabilities to physicians *PH_i_* and *PH_i_* grant capabilities to patient *PA_i_* composed of *GW_i_*_,_*_j_* and *SN_i_*_,_*_j_*_,_*_d_*. Otherwise, The PKG could perform all roles for the granting of capabilities.

The permission hierarchy, as shown in Figure 2, performs a very important role in the FNKAP in two ways that classify the capabilities of each entity in the hierarchy and could help one-round key establishment between two parties in the proposed protocol. Furthermore, note that most of the previous research assumed that the EHR collects the patient's PHI directly by establishing a secure channel between the EHR and the patient and keeps it for further use. After that, physicians log on to the EHR to access the PHI of patient. However, it is possible to expose the privacy data of a patient to the insiders of the EHR in that scenario. For example, in the absence of patient consent, an insider of the EHR may damage the patient's data and harm the patient for their own personal reasons. The FNKAP will cope with the problem by establishing a secure channel between the physician and the patient, not between the EHR and the patient. However, the patient needs to submit his/her PHI to the EHR, because it still needs to take the role of collecting and keeping the PHI from the patient. Thereby, only the legal physician with the proper permission could see the data in the EHR.

### 3.2. System Initialization

Similar to the other IBC-based protocols in [12–14] and the description in Section 2, the FNKAP requires a PKG for the system initialization [9]. Let *k* be the security parameter, *G*_1_ and *G*_2_ be two cyclic groups of prime order *q* and *ê*: *G*_1_ × *G*_1_ → *G*_2_ be a bilinear pairing. Let *G*_1_* be the non-identity element set of *G*_1_. It is assumed that public keys, *i.e.*, identities or amplified identities, at depth *l* are vectors of elements in (*G*_1_*)*^l^*. The *j*-th component corresponds to the identity at level *j*. The system later extends the construction to public keys over {0, 1}* by first hashing each component *I_j_* using a collision-resistant hash *H*(·): {0,1}* → *G*_1_*.

The u-Health server with identity *ID_SV_* creates a private key set (*S*_1_, *S*_2_, *S*_3_, *S*_4_) for a WHMS and computes an amplified identity *AD_SV_* = *H*(*ID_SV_*) and *AD_SV_*·*S*_1_. After that, *SV* stores the information in its memory. The notations used in the FNKAP are listed in Table 1.

### 3.3. Physician and Patient Registration

**Figure 3.**
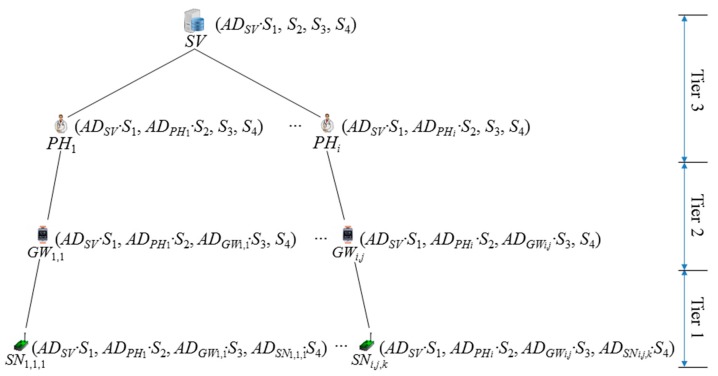
Hierarchical key setup for the freshness-preserving non-interactive hierarchical key agreement protocol (FNKAP).

This phase is so that each participant has a pair of keys for the secure channel establishment based on the IBC. It is assumed that the physician *PH_i_* has a greater power compared to the patient *PA_i_* with a gateway node *GW_i_*_,_*_j_* and some sensor nodes *SN_i_*_,_*_j_*_,_*_d_*. The role of nodes in the hierarchy is pre-allocated before the phase. To allow identity revocation, *SV* could add a random number *r_i_* into *AD_i_*, such that each of the amplified identities is derived as *AD_i_* = *H*(*ID_i_*‖*r_i_*). Figure 3 shows the established key after the system initialization phase and the registration phase for the FNKAP. Each node in the tree contains the bindings between the amplified identity and the secret key of the node and the old entities in its hierarchy.

#### 3.3.1. Physician Registration

To register, a physician *PH_i_* submits his/her identity *ID_PHi_* to the u-Health server *SV*. *SV* first validates the received identity. If validation is successful, it computes *AD_PHi_* = *H*(*ID_PHi_*) and *AD_PHi_*·*S*_2_. After that, *SV* sends {(*AD_SV_*·*S*_1_, *AD_PHi_*·*S*_2_, *S*_3_, *S*_4_) and (*AD_SV_, AD_PHi_*)} to *PH_i_* via a secure channel, and *PH_i_* keeps the information in private, which is preferably stored in the smartcard of his/her identity card or the smart device.

#### 3.3.2. Patient Registration

Patient registration is required for the subscription to the service from the WHMS, which needs to issue some sensor nodes and a gateway, depending on the physician's prescription for the patient *PA_i_*. Let *PA_i_* be a patient seeking medical help from *PH_i_*. Patient registration is only possible after *PA_i_* gets a proper prescription from *PH_i_*. To register, *PA_i_* submits his/her identity *ID_PAi_*, the attending physician's identity *PH_i_*, the prescription for the sensor nodes and a smart device to the u-Health server *SV*. The attending physician's identity could be omitted from *PA_i_*'s registration if *SV* could set the relationship between *PA_i_* and *PH_i_*. *SV* first validates the received identities. Only if the validation is successful does *SV* check the prescription and set up some sensor nodes with a smart device. *SV* needs to issue a smart device if *PA_i_* does not have any smart device, which works as the role of a gateway *GW_i_*_,_*_j_*. *SV* computes *AD_GWi_*_,_*_j_* = *H*(*ID_GWi_*_,_*_j_*), *AD_GWi_*_,_*_j_*·*S*_3_, *AD_SNi_*_,_*_j_*_,_*_d_* = *H*(*ID_SNi_*_,_*_j_*_,_*_d_*) and *AD_SNi_*_,_*_j_*_,_*_d_*·*S*_4_, where *d* is in 1 ≤ *d* ≤ *k* if *PA_i_* requires *k* sensor nodes in the prescription from *PH_i_*. After that, *SV* stores {(*AD_SV_*·*S*_1_, *AD_PHi_*·*S*_2_, *AD_GWi_*_,_*_j_*·*S*_3_, *S*_4_) and (*AD_SV_, AD_PHi_, AD_GWi_*_,_*_j_*)} in *GW_i_*_,_*_j_*'s memory securely. Furthermore, *SV* stores *k* values of {(*AD_SV_*·*S*_1_, *AD_PHi_*·*S*_2_, *AD_GWi_*_,_*_j_*·*S*_3_, *AD_SNi_*_,_*_j_*_,_*_d_*·*S*_4_) and (*AD_SV_, AD_PHi_, AD_GWi_*_,_*_j_, AD_SNi_*_,_*_j_*_,_*_d_*)} in each *SN_i_*_,_*_j_*_,_*_d_*'s memory securely, 1 ≤ *d* ≤ *k*, respectively.

### 3.4. Non-Interactive Key Agreement and Secure Communication

The purpose of this phase is to establish a secure channel by setting up a fresh session key between the sensor node *SN_i_*_,_*_j_*_,_*_d_* of the patient *PA_i_* and the physician *PH_i_* in the WHMS. To establish a secure channel by using a fresh session key *SN_i_*_,_*_j_*_,_*_d_, PH_i_* conducts the following tasks:

Step 1. *SN_i_*_,_*_j_*_,_*_d_* with its private key set (*AD_SV_*·*S*_1_, *AD_PHi_*·*S*_2_, *AD_GWi_*_,_*_j_*·*S*_3_, *AD_SNi_*_,_*_j_*_,_*_d_*·*S*_4_) chooses a random number *r*_1_, computes *R*_1_ = *r*_1_·*AD_SNi_*_,_*_j_*_,_*_d_* and a fresh session key *SK*_1_ = *ê*(*AD_SV_*·*S*_1_, *AD_SV_*′)·*ê*(*AD_PHi_*·*S*_2_, *AD_PHi_*′)·*ê*(*AD_GWi_*_,_*_j_*·*S*_3_, *AD_PHi_*′)·*ê*(*AD_SNi_*_,_*_j_*_,_*_d_*·*S*_4_, *AD_PHi_*′)*^r^*^1^ by using the amplified identity set of the counterpart *PH_i_*, which is (*AD_SV_*′, *AD_PHi_*′). After that, *SN_i_*_,_*_j_*_,_*_d_* senses its data *Data_i_*, computes *M*_1_ = *E_SK_*_1_(*Data_i_*) and *MAC*_1_ = *H*(*SK*_1_‖*R*_1_‖*M*_1_) and sends {*R*_1_, *M*_1_, *AD_SNi_*_,_*_j_*_,_*_d_, MAC*_1_} to the EHR.
Step 2. When *PH_i_* wants to check the health condition of the patient *PA_i_, PH_i_* needs to be authenticated first by the EHR by using one of the previous schemes [12,16,25,26]. Only after the proper authentication, *PH_i_* could access *PA_i_*'s PHI by establishing the session key *SK*_1_′ = *ê*(*AD_SV_*·*S*_1_, *AD_SV_*′)·*ê*(*AD_PHi_*·*S*_2_, *AD_PHi_*′)·*ê*(*AD_PHi_, AD_GWi_*_,_*_j_*′)*^S^*^3^·*ê*(*AD_PHi_, R*_1_)*^S^*^4^ by using the amplified identity set of the counterpart *PA_i_*, which is (*AD_SV_*′, *AD_PHi_*′, *AD_GWi_*_,_*_j_*′, *AD_SNi_*_,_*_j_*_,_*_d_*′). *PH_i_* assures the correctness of the established fresh session key only if the validity check of *MAC*_1_ is successful, by comparing it with *PH_i_*'s computation of *H*(*SK*_1_′‖*R*_1_‖*M*_1_), reads *Data_i_* by decrypting *M*_1_ with *SK*_1_′ and processes this for the further medical treatment of *PA_i_*.

The above scenario assumed that the PHI of *PA_i_* is collected and sent to *PH_i_* by *SN_i_*_,_*_j_*_,_*_d_*, not by *GW_i_*_,_*_j_*. However, if *GW_i_*_,_*_j_* could have more powerful functionality, each entity on *PA_i_* could have a distinctive role, for which the sensor node only collects the PHI and sends it to the gateway, and the gateway communicates with the EHR after the collection and analysis of the PHI from the sensor nodes. In this scenario, we need to modify the steps of this phase focusing on *GW_i_*_,_*_j_*, which is skipped in this paper, due to the similarity with the following scenario.

If *PH_i_* needs to return back a report to *PA_i_, PH_i_* could send a secure message by using the similar steps as the communication with *SN_i_*_,_*_j_*_,_*_d_*. In this case, *PH_i_* needs to communicate with *GW_i_*_,_*_j_* on *PA_i_*, not via the u-Health server nor the EHR. For the process, *PH_i_* and *GW_i_*_,_*_j_* conduct the following further tasks:

Step 3. *PH_i_* with the private key set (*AD_SV_*·*S*_1_, *AD_PHi_*·*S*_2_, *S*_3_, *S*_4_) chooses a random number *r*_2_, computes *R*_2_ = *r*_2_·*AD_PHi_* and a fresh session key *SK*_2_ = *ê*(*AD_SV_*·*S*_1_, *AD_SV_*′)·*ê*(*AD_PHi_*·*S*_2_, *AD_PHi_*′)·*ê*(*AD_PHi_, AD_GWi_*_,_*_j_*′)*^S^*^3^·*ê*(*AD_PHi_, AD_GWi_*_,_*_j_*′)*^S^*^4·^*^r^*^2^ by using the amplified identity set of the counterpart *GW_i_*_,_*_j_* on *PA_i_*, which is (*AD_SV_*′, *AD_PHi_*′, *AD_GWi_*_,_*_j_*′). After that, *PH_i_* computes *M*_2_ = *E_SK_*_2_(*Data_i_*) and *MAC*_2_ = *H*(*SK*_2_‖*R*_2_‖*M*_2_) and sends {*R*_2_, *M*_2_, *AD_GWi_*_,_*_j_*′, *MAC*_2_} to the EHR.
Step 4. When *GW_i_*_,_*_j_* receives the message from *PH_i_*, it could access the message *M*_2_ by establishing the session key *SK*_2_′ = *ê*(*AD_SV_*·*S*_1_, *AD_SV_*′)·*ê*(*AD_PHi_*·*S*_2_, *AD_PHi_*′)·*ê*(*AD_GWi_*_,_*_j_*·*S*_3_, *AD_PHi_*′)·*ê*(*AD_GWi_*_,_*_j_, R*_2_)*^S^*^4^ by using the amplified identity set of the counterpart *PH_i_*, which is (*AD_SV_*′, *AD_PHi_*′). *GW_i_*_,_*_j_* assures the correctness of the established fresh session key only if the validity check of *MAC*_2_ is successful, by comparing it with *PH_i_*'s computation of *H*(*SK*_2_′‖*R*_2_‖*M*_2_), reads *Data_i_* by decrypting *M*_2_ with *SK*_2_′ and responds by following the message from *PH_i_*.

The FNKAP could support secure communication between any two entities in the hierarchy tree without requiring any pre-communication by establishing a fresh session key in them. Furthermore, it could provide convenience to the patient, because he/she could know his/her health condition anywhere and anytime by directly communicating with his/her attending physician.

## 4. Analysis

This section provides the correctness of the FNKAP and provides the security analysis on it. Furthermore, we provide the functionality and the performance analyses by comparing the FNKAP with the related protocols in [10,16,22].

### 4.1. Correctness

Here, we verify the correctness of the session keys of *SK*_1_ and *SK*_1_′ and *SK*_2_ and *SK*_2_′ based on the properties on the bilinear map described in Section 2. First of all, *SK*_1_ and *SK*_1_′ are consistent as follows:

$$\begin{array}{ll} SK_{1} & =\hat{e}\left(AD_{SV}\cdot S_{1},AD_{SV^{\prime }}\right)\cdot \hat{e}\left(AD_{PHi}\cdot S_{2},AD_{PHi\prime }\right)\cdot \hat{e}\left(AD_{GWi,j}\cdot S_{3},AD_{PHi\prime }\right)\cdot \hat{e}{\left(AD_{SNi,j,d}\cdot S_{4},AD_{PHi\prime }\right)}^{r1}\\ & =\hat{e}\left(AD_{SV}\cdot S_{1},AD_{SV^{\prime }}\right)\cdot \hat{e}\left(AD_{PHi}\cdot S_{2},AD_{PHi\prime }\right)\cdot \hat{e}\left(AD_{GWi,j}\cdot S_{3},AD_{PHi\prime }\right)\cdot \hat{e}\left(r_{1}\cdot AD_{SNi,j,d}\cdot S_{4},AD_{PHi\prime }\right)\\ & =\hat{e}\left(AD_{SV}\cdot S_{1},AD_{SV^{\prime }}\right)\cdot \hat{e}\left(AD_{PHi}\cdot S_{2},AD_{PHi\prime }\right)\cdot \hat{e}\left(AD_{GWi,j}\cdot S_{3},AD_{PHi\prime }\right)\cdot \hat{e}\left(R_{1}\cdot S_{4},AD_{PHi\prime }\right)\\ & =\hat{e}\left(AD_{SV}\cdot S_{1},AD_{SV^{\prime }}\right)\cdot \hat{e}\left(AD_{PHi}\cdot S_{2},AD_{PHi\prime }\right)\cdot \hat{e}{\left(AD_{GWi,j},AD_{PHi\prime }\right)}^{S3}\cdot \hat{e}\left(R_{1}\cdot S_{4},AD_{PHi\prime }\right)\\ & =\hat{e}\left(AD_{SV}\cdot S_{1},AD_{SV^{\prime }}\right)\cdot \hat{e}\left(AD_{PHi}\cdot S_{2},AD_{PHi\prime }\right)\cdot \hat{e}{\left(AD_{GWi,j},AD_{PHi\prime }\right)}^{S3}\cdot \hat{e}{\left(R_{1},AD_{PHi\prime }\right)}^{S4}\\ & =\hat{e}\left(AD_{SV}\cdot S_{1},AD_{SV^{\prime }}\right)\cdot \hat{e}\left(AD_{PHi}\cdot S_{2},AD_{PHi\prime }\right)\cdot \hat{e}{\left(AD_{PHi},AD_{GWi,j\prime }\right)}^{S3}\cdot \hat{e}{\left(AD_{PHi},R_{1}\right)}^{S4}\\ & =SK_{1^{\prime }}. \end{array}$$

*SK*_2_ and *SK*_2_′ are consistent as follows:

$$\begin{array}{ll} SK_{2} & =\hat{e}\left(AD_{SV}\cdot S_{1},AD_{SV^{\prime }}\right)\cdot \hat{e}\left(AD_{PHi}\cdot S_{2},AD_{PHi\prime }\right)\cdot \hat{e}{\left(AD_{PHi},AD_{GWi,j\prime }\right)}^{S3}\cdot \hat{e}{\left(AD_{PHi},AD_{GWi,j\prime }\right)}^{S4\cdot r2}\\ & =\hat{e}\left(AD_{SV}\cdot S_{1},AD_{SV^{\prime }}\right)\cdot \hat{e}\left(AD_{PHi}\cdot S_{2},AD_{PHi\prime }\right)\cdot \hat{e}{\left(AD_{PHi},AD_{GWi,j\prime }\right)}^{S3}\cdot \hat{e}{\left(r_{2}\cdot AD_{PHi},AD_{GWi,j\prime }\right)}^{S4}\\ & =\hat{e}\left(AD_{SV}\cdot S_{1},AD_{SV^{\prime }}\right)\cdot \hat{e}\left(AD_{PHi}\cdot S_{2},AD_{PHi\prime }\right)\cdot \hat{e}{\left(AD_{PHi},AD_{GWi,j\prime }\right)}^{S3}\cdot \hat{e}{\left(R_{2},AD_{GWi,j\prime }\right)}^{S4}\\ & =\hat{e}\left(AD_{SV}\cdot S_{1},AD_{SV^{\prime }}\right)\cdot \hat{e}\left(AD_{PHi}\cdot S_{2},AD_{PHi\prime }\right)\cdot \hat{e}\left(AD_{PHi}\cdot S_{3},AD_{GWi,j\prime }\right)\cdot \hat{e}{\left(R_{2},AD_{GWi,j\prime }\right)}^{S4}\\ & =\hat{e}\left(AD_{SV}\cdot S_{1},AD_{SV^{\prime }}\right)\cdot \hat{e}\left(AD_{PHi}\cdot S_{2},AD_{PHi\prime }\right)\cdot \hat{e}\left(AD_{GWi,j}\cdot S_{3},AD_{PHi^{'}}\right)\cdot \hat{e}{\left(AD_{GWi,j},R_{2}\right)}^{S4}\\ & =SK_{2^{\prime }} \end{array}$$

### 4.2. Security Analysis

Although it is important to provide a formal security proof on any cryptographic protocol, the formal security proof of protocols remains one of the most challenging issues for cryptography research. Until now, a simple, efficient and convincing formal methodology for correctness analysis on security protocols is still an important subject of research and an open problem. Because of these reasons, most protocols have been demonstrated with a simple proof. This section follows the security analysis approaches used in [27]. The security analysis is focused on verifying the overall security requirements for the FNKAP, including passive and active attacks, as follows.

#### Proposition 1

The FNKAP provides entity anonymity.

*Proof*: In the FNKAP, the anonymity of the entity is obtained by applying the hash function and is based on the BDH problem. Two phases in the FNKAP, the registration phase and the non-interactive key agreement and secure communication phase, use amplified identities by using the one-way hash function. The u-Health server only gets the real identity of each entity. There is no way for an attacker to know the real identity, even if the attacker could capture the messages {*R*_1_, *M*_1_, *AD_SNi_*_,_*_j_*_,_*_d_, MAC*_1_} and {*R*_2_, *M*_2_, *AD_GWi_*_,_*_j_*′, *MAC*_2_} during the protocol run of the FNKAP.

#### Proposition 2

The FNKAP cannot reveal the private key set or the generated session key to outsiders.

*Proof*: The security of the private key set is based on the combinations of the amplified identities and the secret values. This indicates that an attacker has to know both of them to retrieve the private key set. However, there is no way that the attacker could derive the secret values or the amplified identities from the private key set due to the BDH and the CDH problems. For the concern of revealing the session key *SK*, the attacker needs to have power to analyze and get necessary information from the intercepted messages {*R*_1_, *M*_1_, *AD_SNi_*_,_*_j_*_,_*_d_, MAC*_1_} and {*R*_2_, *M*_2_, *AD_GWi_*_,_*_j_*′, *MAC*_2_}. However, there is no way that the attacker could know the session key due to the BDH and the CDH problems.

#### Proposition 3

The FNKAP provides session key freshness and thereby can prevent from the replay attack.

*Proof*: The random number *r_i_* used to establish the session key in the non-interactive key agreement and secure communication phase guarantees the freshness of the session key. There is no way that an attacker could get any information to know the session key due to the BDH problem. Furthermore, the FNKAP is strong against the replay attack due to the session key freshness support with *MAC_i_* in each message.

#### Proposition 4

The FNKAP is secure against passive attack.

*Proof*: We assume that an attacker is successful if the attacker knows any useful information from the intercepted messages. We show that the probability of success for learning them is negligible due to the difficulty of the underlying mathematical problems, the BDH and the CDH problems.

- The completeness of the FNKAP is already proven by describing the run of the protocol in Section 3.
- If the attacker is passive, all the attacker can gather are the intercepted messages {*R*_1_, *M*_1_, *AD_SNi_*_,_*_j_*_,_*_d_, MAC*_1_} and {*R*_2_, *M*_2_, *AD_GWi_*_,_*_j_*′, *MAC*_2_}. However, it is negligible to find the key related information from them due to the difficulty of the BDH and the CDH problems.

Finally, we could say that the FNKAP is secure against passive attack.

#### Proposition 5

The FNKAP is secure against active attack.

*Proof*: We could argue that an attack from an attacker is successful if the attacker finds the session key *SK_i_* or knows any of the messages *M*_1_ and *M*_2_. Therefore, we will show that the probability of the success of finding them is negligible due to the difficulty of the BDH and the CDH problems.

- The acceptance by all entities means that each *MAC_i_* in the corresponding message is successfully verified. This means that *MAC_i_* is verified successfully by using the correct session key *SK_i_*. If it is the case that entities accept the messages and they continue the session, the probability that the attacker could modify the messages is negligible. Additionally, the only way for the attacker to find the session key or the private key information is to solve the difficulty of the underlying mathematical problems, the BDH and the CDH problems.
- Now, we consider the active attacker with the following cases.
  (1) There is no way that an attacker could get the private key set related to {*S*_1_, *S*_2_, *S*_3_, *S*_4_} due to the difficulty of the BDH and the CDH problems.
  (2) An attacker cannot masquerade as *SN_i_*_,_*_j_*_,_*_d_* nor *GW_i_*_,_*_j_* to cheat *PH_i_*. This is mainly because the attacker cannot generate valid messages without deriving the correct session key *SK_i_*. Furthermore, the attacker could not compute the proper *MAC_i_*, which is required for the verification of the session for the counter party.
  (3) An attacker cannot impersonate *PH_i_* to cheat *SN_i_*_,_*_j_*_,_*_d_* nor *GW_i_*_,_*_j_*. Only the legal physician *PH_i_* could form the legal messages, which need to be properly matched with the information from the counter party in the protocol run. Even if the attacker could pass the verifications at the protocol steps, the attacker still cannot get any useful information from *M*_1_ and *M*_2_, due to the difficulty of the underlying mathematical problems, and cannot generate the consequent valid messages.
  Finally, we could say that the FNKAP is secure against active attack.

#### 4.3. Functionality and Performance Analyses

This sub-section evaluates the functionality and the performance of the FNKAP and provides comparisons with the FNKAP and the related works of Huang *et al.*, in [10], Mtonga in [16] and Lee *et al.*, in [22], as shown in Tables 2 and 3. We only consider the key agreement from both of Huang *et al.*'s schemes and Mtonga's scheme, even if they provide some other security functions.

For the functionality analysis, we consider the freshness of the session key, the privacy only focused on the anonymity, the integrity of transmitted data and the network environment of the protocols. Huang *et al.*'s scheme uses the public-key cryptosystem to secure the PHI, which does not provide the freshness and the integrity. Mtonga's scheme shares the weaknesses the same as in Huang *et al.*'s scheme, even if it is based on the non-interactive identity-based key agreement. In contrast with the two schemes, Lee *et al.*'s protocol satisfies all of the functionalities, but it is impossible to directly apply to the WHMS.

As shown in Table 3, the performance of the key agreement protocol can be approximated in terms of the communicational loads, the space and computation overheads. The number of rounds is considered as a factor for the communicational load. The communication overhead of Huang *et al.*'s scheme is mentioned as ‘-’, because it does not provide the session key agreement. Except for Huang *et al.*'s scheme, all of the other three protocols share this property, because they share the basic mathematical operation for the key agreement.

For the space and computation loads, we consider the memory requirements and the number of basic operations, including the hash function, the public-key operation, the scalar multiplication and the pairing operation. The key agreement protocol demands extra space to keep the required keys and additional information to set up a secure channel. The space and computation overheads are related to the size of the private key set. An entity in the hierarchy tree needs to store a key set of {*S*_1_, *S*_2_, *S*_3_, *S*_4_} with an amplified identity set {*AD_SV_, AD_PHi_, AD_GWi_*_,_*_j_, AD_SNi_*_,_*_j_*_,_*_d_*}. The size of the key set and the amplified identity in Huang *et al.*'s scheme and Mtonga's scheme do not depend on the size of network entities, but depend on two communication entities. However, the FNKAP and Lee *et al.*'s protocol depend on the height of the hierarchical tree. The FNKAP requires a little bit more computational overhead than Lee *et al.*'s, which is for the WHMS, and requires some additional entities for the WSN.

## 5. Conclusions

In this paper, we have proposed a secure and freshness-preserving non-interactive hierarchical key agreement protocol (FNKAP) for the WHMS. The FNKAP is based on the bilinear paring, the IBC and the non-interactive key agreement. To propose a communication-efficient protocol, we proposed a permission hierarchical tree for WHMSs with the consideration of the network requirements. In the FNKAP, each entity in the WHMS is only pseudonymously identified, hence protecting the entity from the negative effects of identity theft, such as fraudulent insurance claims by attackers. The FNKAP allows the patient and his/her physician to establish a secure channel via a session key, which only requires one-round communication and does not require any further interactive communications. The analyses have shown that the FNKAP achieves good security properties and functionalities. However, the performance comparison has shown that the FNKAP has a bit more overhead than the other protocols, due to the support of the required features for the WHMS.

## Figures and Tables

**Table 1.** Notations.

**Notation**	**Description**
*PA_i_*	Patient *i*
*PH_i_*	Attending physician *i* of *PA_i_*
*SV*	u-Health server
*GW_i_*	Gateway *i*
*SN_i_*_,_*_j_*	Sensor node *j* in the *GW_i_*
*ID_i_*	Entity *i*'s identifier
*AD_i_*	Amplified identity of *ID_i_*
(*S*_1_, *S*_2_, *S*_3_, *S*_4_)	Private key set of PKG, *S_i_*∊Z_q_*
*SK*	Session key established between two entities
*r_i_*	Random number
*Data_i_*	Data from *i*
*G*_1_, *G*_2_	Cyclic groups of prime order *q*
*P*	Generator of *G*_1_
*ê*	Bilinear map *G*_1_ × *G*_1_ → *G*_2_
*H*(·)	One way hash function *H*(·) : {0, 1}*→ *G*_1_*
*E_K_*(*M*)	Symmetric key encryption of *M* by using a key *K*
·	Multiplication
‖	Concatenation

**Table 2.** Functionality comparison.

**Function**	**Freshness**	**Privacy**	**Integrity**	**Network Environment**
**Protocol**				
Huang *et al.*'s in [10]	No	Yes	No	WHMS
Mtonga's in [16]	No	Yes	No	WHMS
Lee *et al.*'s in [22]	Yes	Yes	Yes	WSN
FNKAP	Yes	Yes	Yes	WHMS

**Table 3.** Performance comparison. *pr*, private key with a length of at least 160 bits; *id*, amplified identity with a length of at least 128 bits; *hf*, hash function; *pu*, public-key operation; *sc*, scalar multiplication; *pa*, pairing operation.

**Feature**	**Communication**	**Space**		**Computation**
**Protocol**		**Private Key**	**Amplified Identity**	
Huang *et al.*'s in [10]	-	2*pr*	2*id*	4*pu*
Mtonga's in [16]	1 round	2*pr*	2*id*	1*sc* + 2*pa*
Lee *et al.*'s in [22]	1 round	3*pr*	3*id*	2*hf* + 5*sc*+ 6*pa*
FNKAP	1 round	4*pr*	4*id*	2*hf* + 7*sc* + 8*pa*
